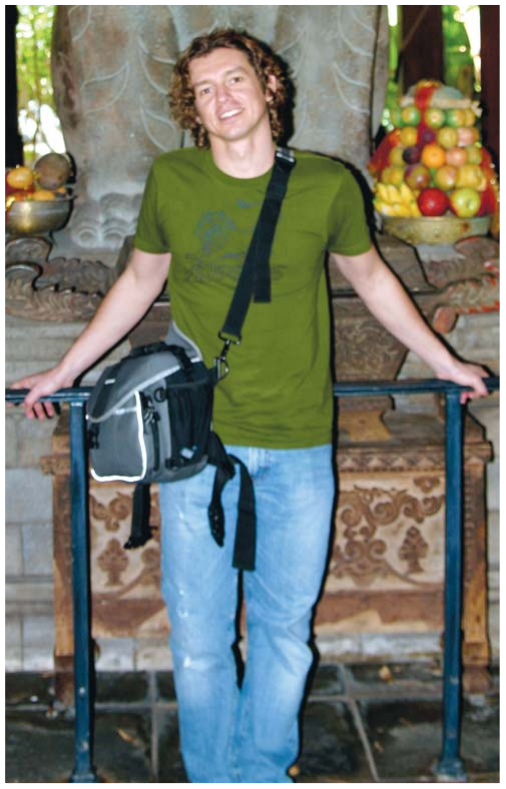# In Memoriam: Christopher G. Reuther, 1973–2007

**Published:** 2007-06

**Authors:** HELEN KELLER

If you’ve ever flipped through this journal and been stopped by a compelling image that you had to examine or an intriguing layout that drew you into an article you might otherwise have skipped, chances are good that it was due to the talents of our colleague and friend Christopher Reuther. For 11 years, Chris created news stories, images, and layouts for *EHP*. His sudden death on April 24 at the age of 34 has left a void in our staff and in the world of environmental health science journalism.

Life is either a daring adventure or nothing. To keep our faces toward change and behave like free spirits in the presence of fate is strength undefeatable.

Chris had an enviable intellect and insatiable curiosity about the world around him. Following a year of college as an engineering student, he transferred to the University of North Carolina at Chapel Hill to pursue a double major in environmental science and journalism—although he loved the mental challenge of very technical problems, Chris was a communicator at heart. His UNC advisor told him that the combination of the two degrees wouldn’t yield him good career opportunities. Undaunted, he applied to both programs, was accepted to each, and completed the degrees simultaneously. Although it’s true that Chris never got rich doing what he did, he found great personal rewards in his work on *EHP*.

Chris began at *EHP* as a student volunteer seeking to learn real-life science journalism. Following graduation, he continued to work at *EHP* first as a writer and later as a contract graphic artist and photographer. During this time he discovered a true love for environmental news reporting. *EHP* ’s mission—to improve the lives of people by enlightening the world to their situations, and by empowering them to improve and sustain their own environment—became his mission.

Chris’s contributions to our success in this mission are numerous. He was uniquely capable of sifting through scientific content to lift out the most salient facts, and then translating that information into images and layouts that went far beyond what text alone could accomplish in helping the reader to understand it, and more importantly to realize its relevance both globally and personally. He was particularly passionate about conveying the situations of those whose voices are not always heard—the poor, the minorities, the very young and the very old—and he did so with utmost respect, sensitivity, and empathy.

In *EHP* ’s weekly news graphics meetings, he would argue passionately for a concept, then seconds later have us laughing out loud at his wickedly clever—and often irreverent—suggestions for headlines. He understood on a visceral level that caring about environmental health issues is as much an experience of joy in the world as fear of it.

Although Chris was preparing to leave *EHP* to embark on a career in environmental law, he never stopped believing in the importance of the journal’s work. Whether creating a two-inch-square photo of a lab mouse or a 10-page layout on global warming, Chris reminded us constantly that life is about attention to the details. And although he brought a keen focus to whatever task was at hand, it was always people that he was most eager, and worked hardest, to portray. His idea of how people should be treated was apparent not only in how Chris worked, but integrally and emphatically in the way he lived. For us, we learned to look at the world just a bit differently because of Chris, and we are the poorer for the loss of his perspective.

To see more of Chris’s work, visit http://www.brogan.com/chris.

## Figures and Tables

**Figure:**